# Cytoreductive surgery plus hyperthermic intraperitoneal chemotherapy with lobaplatin and docetaxel improves survival for patients with peritoneal carcinomatosis from abdominal and pelvic malignancies

**DOI:** 10.1186/s12957-016-1004-4

**Published:** 2016-09-15

**Authors:** Hai-Tao Wu, Xiao-Jun Yang, Chao-Qun Huang, Jian-Hua Sun, Zhong-He Ji, Kai-Wen Peng, Qian Zhang, Yan Li

**Affiliations:** 1 Department of Peritoneal Cancer Surgery, Beijing Shijitan Hospital Affiliated to the Capital Medical University, No 10 Tieyi Road, Yangfangdian, Haidian District, Beijing 100038 China; 2 Department of Oncology, Zhongnan Hospital Wuhan University, Hubei Key Laboratory of Tumor Biological Behaviors and Hubei Cancer Clinical Study Center, Wuhan, 430071 China

**Keywords:** Lobaplatin, Docetaxel, Peritoneal carcinomatosis, Cytoreductive surgery, Hyperthermic intraperitoneal chemotherapy

## Abstract

**Background:**

This work was to evaluate the perioperative safety and efficacy of cytoreductive surgery (CRS) plus hyperthermic intraperitoneal chemotherapy (HIPEC) with lobaplatin and docetaxel in patients with peritoneal carcinomatosis (PC) from gastrointestinal and gynecological cancers.

**Methods:**

Patients were treated by CRS + HIPEC with lobaplatin 50 mg/m^2^ and docetaxel 60 mg/m^2^ in 6000 mL of normal saline at 43 ± 0.5 °C for 60 min. Vital signs were recorded for 6 days after CRS + HIPEC procedures. Perioperative serious adverse events (SAE), hematological, hepatic, renal, and electrolytes parameters, the changes in serum tumor markers (TM) before and after operation, patient recovery, and overall survival (OS) were analyzed.

**Results:**

One hundred consecutive PC patients underwent 105 CRS + HIPEC procedures and postoperative chemotherapy. The median CRS + HIPEC duration was 463 (range, 245–820) min, and the highest temperature and heart rate during six postoperative days were 38.6 °C (median 37.5 °C) and 124 bpm (median 100 bpm), respectively. The 30-day perioperative SAE occurred in 16 (15.2 %) and mortality occurred in 2 (1.9 %) patients. Most routine blood laboratory tests at 1 week after surgery turned normal. Among 82 cases with increased preoperative TM CEA, CA125, and CA199, 71 cases had TM levels reduced or turned normal. Median time to nasogastric tube removal was 5 (range, 3–23) days, to liquid food intake 6 (range, 4–24) days, and to abdominal suture removal 15 (range, 10–30) days. At the median follow-up of 19.7 (range, 7.5–89.2) months, the median OS was 24.2 (95 % CI, 15.0–33.4) months, and the 1-, 3-, and 5-year OS rates were 77.5, 32.5, and 19.8 %, respectively. Univariate analysis identified five independent prognostic factors on OS: the origin of PC, peritoneal cancer index, completeness of CRS, cycles of adjuvant chemotherapy, and SAE.

**Conclusions:**

CRS + HIPEC with lobaplatin and docetaxel to treat PC is a feasible procedure with acceptable safety and can prolong the survival in selected patients with PC.

**Trial registration:**

ClinicalTrials.gov, NCT00454519

## Background

The locoregional progression of gastrointestinal and gynecological malignancies such as gastric cancer (GC), colorectal cancer (CRC), ovarian cancer (OC), primary peritoneum carcinomatosis (PPC), pseudomyxoma peritonei (PMP), malignant peritoneal malignancies (MPM), frequently results in peritoneal carcinomatosis (PC), characterized by the presence of tumor nodules of various size, number, and distribution on the peritoneal surface, with significant negative impact on both survival and quality of life, due to refractory ascites, progressive intestinal obstruction, and unrelieved abdominal pain. The traditional therapies for PC include systemic chemotherapy, palliative surgery, and best support care, without any hope of cure. PC patients have a very poor prognosis with median survival less than 6 months [1, 2].

With better understanding of the tumor biological behaviors and advances in treatment technologies, the landscape of PC has changed remarkably, in that PC is regarded as locoregional disease rather than widespread terminal condition. Over the past decades, novel therapeutic approaches to PC have emerged, combining cytoreductive surgery (CRS) plus hyperthermic intraperitoneal chemotherapy (HIPEC), which integrate the advantages of surgically removing the bulky visible tumor burden and regional hyperthermic chemotherapy eradicating micrometastases and invisible free cancer cells [3].

Hyperthermia and chemotherapy have synergistic effects, which is significantly enhanced at 42 °C [4–6]. The chemotherapeutic regimens for HIPEC include cisplatin, carboplatin, oxaliplatin, fluorouracil, mitomycin, paclitaxel and docetaxel, among others [7–9]. A study of antitumor drugs has been conducted because of the success of cisplatin. Forty years after the discovery of the biological activity of cisplatin for the first time, oxalipaltin and carboplatin as routine chemotherapeutics are widely used in clinical application today, while nedaplatin, heptaplatin, and lobaplatin have only been authorized, respectively, in Japan, South Korea, and China [10]. Though the response rate for first-line carboplatin and paclitaxel is 70 to 80 %, this approach still yields poor results and overall 5-year survival rate is less than 30 % [8]. Lobaplatin is a third-generation platinum compound, which has demonstrated various advantages, including potent antineoplastic activity, no significant nephrotoxicity or neurotoxicity [11], no cross-resistance with cisplatin [12], and relative molecular mass larger than other platinum drugs with the advantage of pharmacokinetics in intraperitoneal chemotherapy. Docetaxel is a semisynthetic compound in the taxane class of anticancer drugs. Although thought to operate through the same mechanism of action as paclitaxel by limiting microtubule depolymerization, the anti-microtubule depolymerization capacity of docetaxel is twice as much as paclitaxel. Moreover, docetaxel has been found to have different spectra of activity and incomplete cross-resistance.

Phase I/II clinical trials of lobaplatin in combination with docetaxel have demonstrated acceptable safety and efficacy for a variety of malignant solid tumors [13, 14]. Several basic researches have suggested that lobaplatin and docetaxel prove synergistic antineoplastic effects when used in combination [15]. Therefore, we summarize the clinical results of CRS + HIPEC with lobaplatin and docetaxel to treat PC at our center.

## Methods

### Patient selection

This study included 100 consecutive patients of PC treated by 105 CRS + HIPEC procedures from January 2008 to June 2015, including 41 patients from CRC, 30 from GC, 16 from OC and PPC, and 13 from PMP. Major clinicopathological characteristics of the patients are listed in Table 1. The evaluations and major inclusion and exclusion criteria were reported previously [16].

**Table 1:** Major clinicopathologic characteristics of the 100 PC patients

Items	Value, *n* (%)
Gender	
Male/female	41 (41)/59 (59)
Age (years)	
<60/≥60	71 (71)/29 (29)
Median KPS score (range)	70 (60–90)
Primary tumor	
Carcinoma of the stomach	30 (30)
Carcinoma of the colorectum	41 (41)
Pseudomyxoma peritonei	13 (13)
Carcinoma of ovary and primary peritoneum	16 (16)
Histopathology	
Adenocarcinoma, poorly/intermediately differentiated	44 (44)
Undifferentiated carcinoma	3 (3)
Myxoadenocarcinoma and signet-ring cell carcinoma	26 (26)
Serous adencarcinoma	14 (14)
Pseudomyxoma peritonei	13 (13)
Neoadjuvant chemotherapy	
Yes/no	47 (47)/53 (53)
PC timing^a^	
Synchronous/metachronous	80 (80)/20 (20)
PCI scores^a^	
≤20/>20	44 (44)/56 (56)
Median PCI scores (range)	22 (3–39)
Ascites at surgery^b^	
≤1000 mL/>1000 mL	63 (63)/37 (37)
Surgical procedures-organ resection	
Partial/total gastrectomy	24 (24)
Resection of jejunum	4 (4)
Resection of ileum	12 (12)
Resection of ileocecus	32 (32)
Ascending colectomy	27 (27)
Transverse colectomy	32 (32)
Descending colectomy	12 (12)
Sigmoidectomy	16 (16)
Rectectomy	29 (29)
Resection ovarian/fallopian tube	29 (29)
Hysterectomy	15 (15)
Partial hepatectomy	2 (2)
Splenectomy	11 (11)
Cholecystectomy	11 (11)
Number of organ resected^a,c^	
1–3 resections	59 (67.1)
4–7 resections	23 (26.1)
8–12 resections	6 (6.8)
Peritonectomy^a^	
Greater/lesser omentum	92 (92)
Left diaphragmatic copula	15 (15)
Right diaphragmatic copula	38 (38)
Right colon gutter	41 (41)
Left colon gutter	42 (42)
Liver round ligament/sickle ligament	53 (53)
Douglas/rectovesical pouch	49 (49)
Anterior wall peritoneum	40 (40)
Pelvic peritoneum	58 (58)
Mesenteric fulguration	66 (66)
Peritoneal resection area^a^	
1–3 resections	44 (44)
4–6 resections	16 (16)
7–12 resections	40 (40)
CC scores^a^	
0	30 (30)
1	32 (32)
2–3	38 (38)
Number of anastomosis^a^	
None or ostomy only	25 (25)
=1/>1	35 (35)/40 (40)
Postoperative chemotherapy mode^d^	
SC/SC + IP	31 (38.3)/50 (61.7)
Postoperative chemotherapy cycles	
<6/≥6	49 (49)/51 (51)
Median postoperative chemotherapy cycles (range)	6 (1–12)
Median postoperative SC/IP + SC cycles (range)^d^	5 (1–12)/6 (1–12)
Median duration of ICU (hours) (range)	22 (11–94)
Median duration of hospitalization (days) (range)	20 (10–76)
Median follow-up (months) (range)	19.7 (7.5–89.2)

^a^According to the first surgery

^b^Five patients each underwent two operations

^c^12 patients without organ resection

^d^19 patients without any postoperative chemotherapy

*PC* peritoneal carcinomatosis, *KPS* Karnofsky performance score, *PCI* peritoneal cancer index, *CC* completeness of cytoreduction, *SC* systemic chemotherapy, *IP* intraperitoneal chemotherapy, *ICU* intensive care unit

### CRS + HIPEC procedure

A designated team of surgical oncologists, anesthesiologists, and operating room staff focusing on PC therapy performed CRS + HIPEC procedure, with detailed description reported previously [7, 16]. Before being anesthetized, the Expression Sequential Compression System Vascular Refill Detection device was wrapped to both legs of the patient in order to prevent deep venous thrombosis because of longer operation time. The abdominal exploration was conducted under general anesthesia and stable hemodynamic monitoring, through a midline xyphoid-pubic incision. Once the abdominal wall was open, detailed assessment of PC was carried out, recording the size and distribution of tumor nodules, and the quantity and quality of ascites, to calculate the peritoneal cancer index (PCI) according to the principle by Sugarbaker [17]. Then maximal CRS was conducted to remove the primary and metastatic tumor with acceptable margins, any involved adjacent structures, regional lymph nodes, and peritonectomy where peritoneal surfaces were involved by tumor [17]. The completeness of cytoreduction (CC score) was determined according to Sugarbaker’s criteria [18] before HIPEC. CC-0 indicates no residual peritoneal disease after CRS; CC-1 represents less than 2.5 mm of residual disease; CC-2 means residual tumor between 2.5 mm and 2.5 cm; and CC-3 indicates more than 2.5 cm of residual tumor or the presence of a sheet of unresectable tumor nodules.

After CRS, abdominal cavity was sufficiently open by Mediflex Surgical Products and all-layer surgical incision protector covered the open abdomen in order to keep the temperature stable and avoid chemotherapy drugs diffusing into the air before HIPEC, because the open coliseum technique was thought to provide optimal thermal homogeneity and spatial diffusion [17], with 50 mg/m^2^ of lobaplatin and 60 mg/m^2^ of docetaxel each dissolved in 3 L of saline. When the perfusion saline was kept at 43.0 ± 0.5 °C and monitored with temperature sensors on real time by an automatic hyperthermia chemotherapy perfusion device (ES-6001, Wuhan E-sea Digital Engineering, Wuhan, China), the perfusion solution was infused at a rate of 400 mL/min. The total HIPEC time was 60 min. After HIPEC, the perfusion solution in the peritoneal cavity was withdrawn by the suction. Then the operation field was checked again for any suspected mishaps. The anastomoses were made to restore the continuity of the digestive tract. Routine colostomy was not performed, but in patients with extensive pelvic resections of the sigmoid colon, rectum, uterus, and bladder, colostomy was made. Then drainage tubes were placed at appropriate sites of anastomosis and depending on the type of primary operation. The incision was closed with a tension-reduced suture, and patient was transferred to the intensive care unit (ICU) for recovery.

### Postoperative monitoring

All patients were carefully monitored for the following parameters: (1) The changes in body temperature and heart rate were recorded for 6 days after surgery; (2) The complete peripheral blood routine, blood biochemical parameters were examined on the first and seventh postoperative days, and cardio-pulmonary function was monitored, the change in serum tumor markers (TM) before and after operation; (3) Anastomotic leakage, intra-abdominal infection, hemorrhage, intestinal obstruction, and other life-threatening serious adverse events (SAE); (4) Wound healing, including fat liquefaction, incision split, surgical site infection, incisional hernia, time to suture, and drainage tube removal; (5) Postoperative recovery of gastrointestinal and other organs functions, including bowel sound, flatus passage, defecation, time of removing nasogastric tube and urinary catheter, and liquid food intake time; and (6) Survival outcomes.

### Postoperative chemotherapy and follow-up

Postoperative adjuvant chemotherapy included intraperitoneal chemotherapy (IP) through the IP port mostly using lobaplatin (50 mg/m^2^, on day 1, every 21 days) and docetaxel (60 mg/m^2^, on day 1, every 21 days), and systemic chemotherapy (SC) mainly with irinotecan, leucovorin, and 5-fluorouracil (FOLFIRI) and oxaliplatin, leucovorin, and 5-fluorouracil (FOLFOX) regimens, all dosed according to body surface area calculation [17, 19]. Platinum combined with docetaxel was used in IP as had been proved to improve OS for PC patients with acceptable safety [20]. IP were given after the patients fully recovered from the operation, and SC was delivered with IP synchronously or alternately. Chemotherapy information was listed in Table 1.

All patients underwent routinely postoperative follow-up with physical examination, chest and abdominopelvic computed tomography, full gastrointestinal contrast media, and serum tumor markers (TM) every 3 months during the first 2 years and then every 6 months thereafter by outpatient consultation or by telephone. The last follow-up was on June 8, 2015, and the overall follow-up rate was 100 %.

### Statistical analysis and term definition

All data were obtained from a prospectively established database of clinical records, surgical and pathology reports, image examination and laboratory reports, and follow-up records. And data were analyzed by SPSS software for windows, version 19.0 (SPSS Inc., Chicago, IL, USA), with *P* < 0.05 considered as statistically significant. The numerical data were directly recorded, and the category data were recorded into different categories.

The following study parameters were defined: (1) Perioperative period: from the day of CRS + HIPEC to day 30 postoperation; (2) Metachronous PC: after the primary tumor had been treated, the patients developed PC during follow-up; (3) Synchronous PC: PC was diagnosed synchronously at first treatment; (4) PCI ≤20 was defined as low PCI (LPCI), and PCI >20 was defined as high PCI (HPCI) [18]; (5) The current research defined CC0-1 as complete cytoreduction and CC2-3 as incomplete cytoreduction [18]; (6) Adverse events: the CRS + HIPEC related complications during the perioperative period, including SAE and other side effects; the former mainly involved life-threatening complications such as intestinal or anastomotic leakage, intestinal obstruction, hemorrhage, and sepsis, and the latter included of anemia, hepatic and renal toxicity, electrolyte disturbance, hypoalbuminemia, myelosuppression, and delayed wound healing, all on the basis of NCI Common Terminology Criteria (CTC) for Adverse Events version 4.0 [21]; (7) Overall survival (OS): the time period from CRS + HIPEC to death as a result of disease for metachronous PC; and the time period from first treatment of PC to death due to synchronous PC.

## Results

A total of 100 PC patients were treated with 105 CRS + HIPEC procedures, including five patients each receiving two CRS + HIPEC procedures (all of five patients due to tumor recurrence). There were 59 women and 41 men, with a median age of 51 (range, 23–73) years. The median operation time was 463 (range, 245–820) min. During surgery, the median volume of blood loss, ascites, and urine output were 600 (range, 100–4000) mL, 500 (range, 0–4200) mL, and 1200 (range, 200–3350) mL, respectively, and the median transfusion volume of red blood cells, plasma, cryoprecipitation and other fluids were 600 (range, 0–3600) mL, 600 (range, 0–2350) mL, 150 (range, 0–300) mL, and 3500 (range, 300–13,500) mL, respectively. In terms of organ resection, 59 patients received removal of 1-3 organs segments, 23 patients had excisions involving 4-7 organs segments, and 6 patients had resections involving 8–12 organs segments. All patients underwent peritonectomy in more than one area of the peritoneal and pelvic cavity (Table 1) and stayed in ICU a median of 22 (range, 11–94) h.

Wound healing was successful in all but three patients who had fat liquefaction. The median time to urinary catheter removal was 3 (range, 2–6) days, to nasogastric tube removal 5 (range, 3–23) days, to fluid food intake 6 (range, 4–24) days, to drainage tube removal 9 (range, 5–69) days, to abdominal sutures removal 15 (range, 10–30) days, and to hospital discharge 20 (range, 10–76) days.

After operation, 30 patients received a median of 5 (range, 1–12) cycles of SC and 51 patients received a median of 6 (range, 1–12) cycles of PIC + SC procedures. The other patients did not receive adjuvant chemotherapy due to intestinal leakage (*n* = 4), intestinal obstruction (*n* = 2), acute myocardium infraction (*n* = 1), and declining any chemotherapy (*n* = 12).

### Vital signs during perioperative period

The body temperature and heart rate decreased gradually to normal in a week after CRS + HIPEC, the number of patients with body temperature exceeding 37 °C were 95, 57, 11, and 1 during the first 4 days after operation (Table 2).

**Table 2:** Important monitoring data of 100 PC patients with 105 CRS + HIPEC procedures during the 6 days after surgery

Index	Range (median)					
	Day 1	Day 2	Day 3	Day 4	Day 5	Day 6
Temperature (°C)	36.4–38.6 (37.5)	36.4–38.0 (37.0)	36.2–37.5 (36.5)	36.2–37.3 (36.5)	36.2–37.0 (36.5)	36.2–36.8 (36.5)
Heart rate (bpm)	76–124 (100)	65–120 (84)	62–120 (80)	60–90 (75)	60–84 (72)	60–84 (72)

### Laboratory results during perioperative period

The major laboratory results are listed in Table 3. One week before CRS + HIPEC, TM increased in 82 (78.1 %) of 105 CRS + HIPEC procedures, including cancer antigen 125 (CA125) only increased in 23, carcinoembryonic antigen (CEA) only increased in nine, and cancer antigen 199 (CA199) only increased in three; CEA + CA125 increased in nine, CEA + CA199 increased in nine, CA125 + CA199 increased in ten, and all these three increased in 19. One week after CRS + HIPEC, CEA, CA125, CA199, CEA + CA125, CEA + CA199, CA125 + CA199, and CEA + CA125 + CA199 decreased or returned to normal in 9, 21, 3, 9, 7, 5, 17, respectively, and all of that in 71.

**Table 3:** Blood profile and biochemical test results of 100 PC patients with 105 CRS + HIPEC procedures

Parameter	Median (range)		Normal value
	Day 1	Day 7	
Peripheral blood test			
Hemoglobin (g/L)	112.5 (66.0–155.0)	116.6 (84.5–151.0)	120–160
Red blood cell (×10^9^/L)	3.96 (2.21–5.55)	3.9 (3.04–4.99)	4–5.5
White blood cell (×10^9^/L)	9.37 (1.33–21.97)	7.74 (4.10–15.60)	4–10
Neutrophil count (×10^9^/L)	8.6 (0.83–20.93)	5.73 (2.64–12.80)	2–7
Platelet count (×10^9^/L)	177 (58–459)	213 (93–555)	100–300
Liver function tests			
Aspartate aminotransferase (U/L)	37 (8–310)	13–51 (22)	0–46
Alanine aminotransferase (U/L)	36 (11–163)	20 (11–50)	0–46
Total bilirubin (μmol/L)	16.4 (4.5–56.6)	12.6 (5.5–24.8)	0–25
Direct bilirubin (μmol/L)	5.2 (0.3–38.2)	3.4 (0.1–11.1)	0–7
Indirect bilirubon (μmol/L)	10.5 (2.3–40.0)	9.5(3.3–20.0)	1.5–18
Total protein (g/L)	50.1 (31.9–67.2)	62.5 (46.8–75.8)	60–80
Albumin (g/L)	26.1 (10.8–44.3)	29.7–47.2 (38.6)	35–55
Globulin (g/L)	24 (10.4–35.1)	23 (13.8–36.6)	20–30
Gamma glutamyl transferase (g/L)	17 (5–132)	40 (10–154)	5–55
Alkaline phosphatase (U/L)	56 (11–135)	76 (36–154)	35–134
Renal function tests			
Blood urea nitrogen (mmol/L)	3.7 (0.8–8.0)	4.8 (1.7–10.3)	1.7–7.2
Creatine (μmol/L)	50.5 (22.0–91.9)	56.9 (30.1–97.1)	45–117
Electrolytes			
K^+^ (mmol/L)	3.68 (3.05–4.84)	4.35 (3.52–5.45)	3.5–5.5
Na^+^ (mmol/L)	136.4 (125.6–155.5)	138 (132.1–144.0)	135–145
Cl^-^ (mmol/L)	102.3 (93.4–122.4)	100.9 (96.1–113.0)	96–106
Ca^2+^ (mmol/L)	1.91 (1.51–2.33)	2.15 (1.91–2.69)	2–2.7
Mg^2+^ (mmol/L)	0.85 (0.64–1.14)	0.96 (0.81–1.15)	0.85–1.15

### Survival analysis

**Fig. 1:**
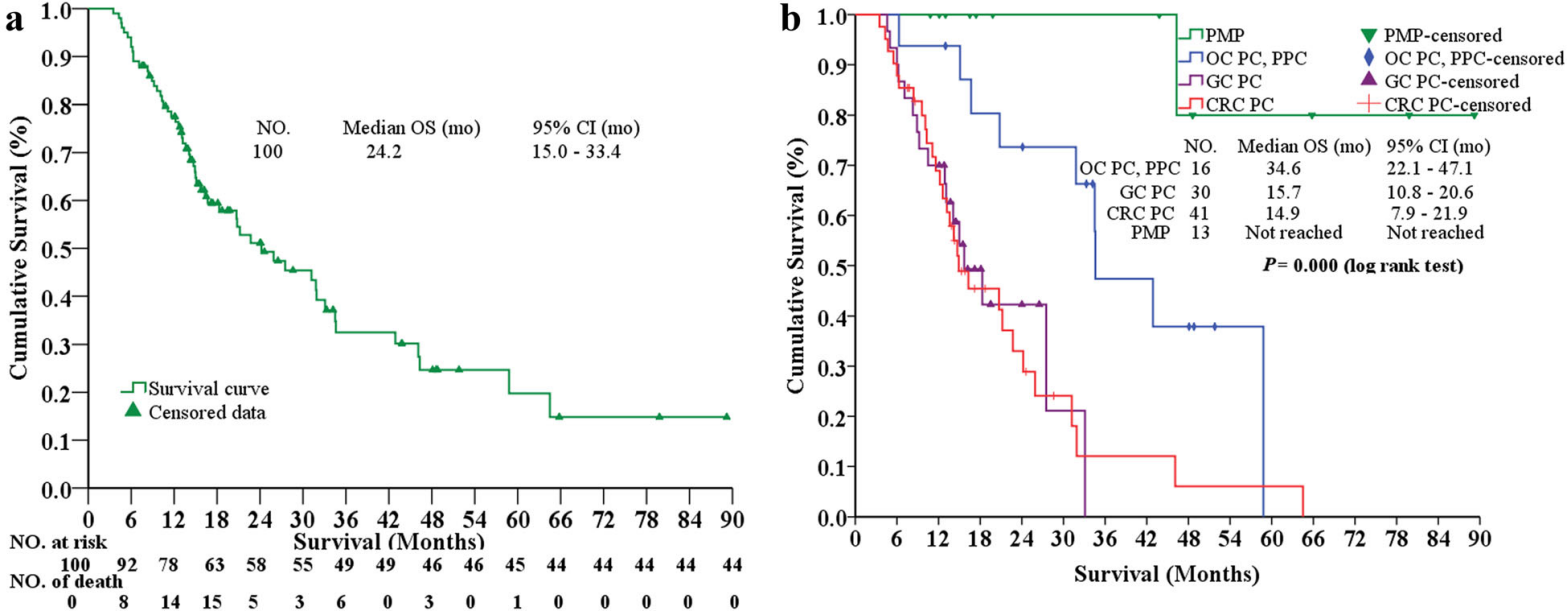
The Kaplan-Meier OS curves for the whole patients treated by CRS + HIPEC (**a**), and comparisons of OS among different primary diseases (**b**). *mo* months, *OC* ovarian cancer, *PPC* primary peritoneum carcinomatosis, *GC* gastric cancer, *CRC* colorectal cancer, *PMP* pseudomyxoma peritonei, *CRS* cytoreductive surgery, *HIPEC* hyperthermic intraperitoneal chemotherapy

**Fig. 2:**
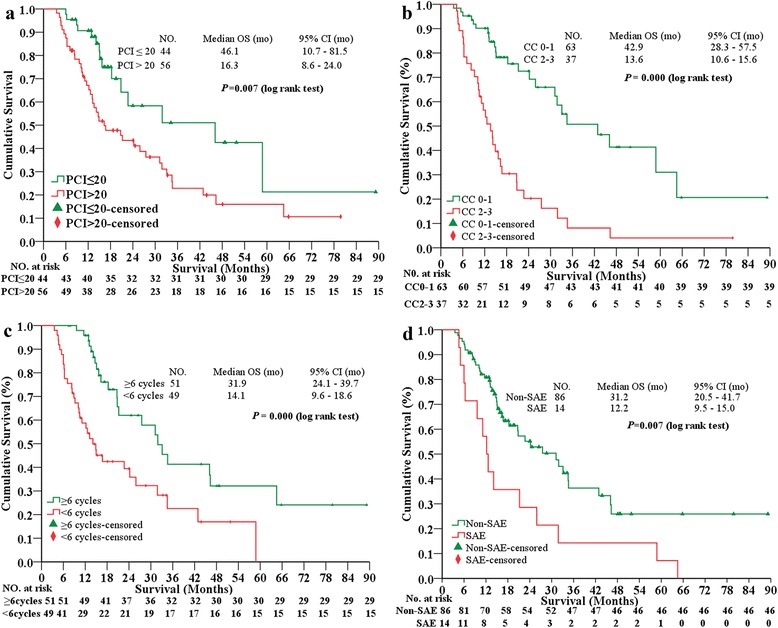
Kaplan-Meier survival curves. The statistical significance in overall survival (OS) comparisons stratified by PCI (**a**), CC (**b**), postoperative adjuvant chemotherapy (**c**), and SAE (**d**). *mo* months, *PCI* peritoneal cancer index, *CC* completeness of cytoreduction, *SAE* serious adverse events

At the median follow-up of 19.7 (range, 7.5–89.2) months for all PC patients in this study, there were 55 (55 %) patients who died, 16 (16 %) survived with tumor, and 29 (29 %) survived without tumor. The 1-, 3-, 5-year OS rates were 77.5, 32.5, and 19.8 %, respectively. The median OS was 24.2 (95 % CI, 15.0–33.4) months (Fig. 1a). The OS comparisons were stratified based on major clinicopathological factors (Table 4). Female patients, age ≥60 years, non-neoadjuvant chemotherapy, synchronous PC, and ascites ≤1000 mL were factors for better OS. The OS of PC patients from PMP, OC + PPC, GC and CRC were not reached, 34.6 (95 % CI, 22.1–47.1) months, 15.7 (95 % CI, 10.8–20.6) months, and 14.9 (95 % CI, 7.9–21.9) months (*P* = 0.000, log rank test) (Fig. 1b), respectively. The median OS for LPCI *vs.* HPCI was 46.1 (95 % CI, 10.7–81.5) *vs.* 16.3 (95 % CI, 8.6–24.0) months (*P* = 0.007, log rank test) (Fig. 2a). The median OS for CC0-1 *vs.* CC2-3 was 42.9 (95 % CI, 28.3–57.5) *vs.* 13.6 (95 % CI, 10.6–15.6) months (*P* = 0.000, log rank test) (Fig. 2b). The median OS for postoperative chemotherapy ≥6 *vs. *<6 cycles was 31.9 (95 % CI, 24.1–39.7) months *vs.* 14.1 (95 % CI, 9.6–18.6) months (*P* = 0.000, log rank test) (Fig. 2c). The median OS for non-SAE *vs.* SAE was 31.2 (95 % CI, 20.5–41.9) months *vs.* 12.2 (95 % CI, 9.5–15.0) months (*P* = 0.007, log rank test) (Fig. 2d).

**Table 4:** OS comparisons stratified by major clinicopathological factors

Variables	Number	Median OS (mo)	95 % CI (mo)	*P*
Gender				0.955
Male	41	22.7	6.2–39.2	
Female	59	24.2	14.2–34.2	
Age (year)				0.608
<60	71	22.7	13.4–32.0	
≥60	29	25.9	7.0–44.8	
Primary tumor				0.000
Carcinoma of the stomach	30	15.7	10.8–20.6	
Carcinoma of the colorectum	41	14.9	7.9–21.9	
Pseudomyxoma peritonei	13	Not reached	Not reached	
Carcinoma of ovary and primary peritoneum	16	34.6	22.1–47.1	
Neoadjuvant chemotherapy				0.128
No	53	31.2	18.4–44.0	
Yes	47	20.7	11.6–29.8	
PC timing				0.086
Synchronous	80	27.5	18.1–36.9	
Metachronous	20	13.2	10.6–15.8	
PCI scores				0.000
≤20	44	46.1	10.7–81.5	
>20	56	16.3	8.6–24.0	
CC scores				0.000
0–1	63	42.9	28.3–57.5	
2–3	37	13.6	10.6–15.6	
Postoperative chemotherapy cycles				0.000
<6	49	14.1	9.6–18.6	
≥6	51	31.9	24.1–39.7	
SAE				0.007
No	86	31.2	20.5–41.9	
Yes	14	12.2	9.5–15.0	
Ascites				0.095
≤1000 mL	63	24.2	13.5–34.9	
>1000 mL	37	21.2	4.8–37.7	

In the original surgery calculation

*OS* overall survival, *mo* months

### Serious adverse events (SAE)

SAE (grades 3–5) occurred in 16 (15.2 %) of 105 CRS + HIPEC procedures (Table 5). Five patients developed gastrointestinal obstruction, four gradually recovered by active conservation remedy, and one with severe gastroplegia returned to normal gastrointestinal function 13 days after surgery. Four patients developed intestinal leakage. The first patient with generalized peritonitis syndrome on postoperative day 4 received a reoperation to repair the anastomosis on postoperative day 10 but failed to repair the leakage and then turned to conservative treatment, the patient survived 2.2 months after the surgery. The second patient developed serious gastric-jejunum anastomosis fistula and sigmoid-rectum anastomosis fistula on postoperative day 8, generalized peritonitis, peritoneal abscess formation and septicemia because of *Staphylococcus aureus* and *Candida tropicalis* infection, with flushing abdominal cavity, intraperitoneal drainage, antibiotics, and total parenteral nutrition support, the patient survived 2.3 months after the operation. The third patient with generalized peritonitis syndrome on postoperative day 11 then developed septicemia due to gram-negative bacilli; given the above active conservative treatment, the patient survived 24 days after the surgery. The fourth patient developed late-onset mild anastomosis fistula on postoperative day 30 and received conservative treatment; the patient survived 3 months after the surgery.

**Table 5:** Detailed information on 16 cases with SAE

Events	Gender/age (year)	Primary tumor	PC time	PCI	Resection		No. Anastomosis	CC	SAEs	Intervention^c^	OS (month)
					Organ	Peritoneal					
Intestinal obstruction (*n* = 5, 4.8 %)	F/45	Colon	Met	24	2	9	1	1	CIO/AI	CT	64.5, D
	M/40	Colon	Met	23	0	3	0	2	IIO/AI	CT	12.2, D
	F/23	Stomach	Syn	30	1	2	2	2	CIO/AI	CT	2.3, D
	F/52	Colon	Syn	15	0	8	0	1	IIO/AI	CT	58.8, S
	F/31	Colon	Met	24	5	9	1	1	CIO/AI and combined gastroplegia	CT	21.2, D
Intestinal leakage (*n* = 4, 3.8 %)	F/26	Colon	Syn	33	2	3	1	3	Limited peritonitis syndrome	CT	12.6, D
	F/40^a^	Stomach	Syn	28	5	9	3	2	*Staphylococcus aureus* and fungus infection, generalized peritonitis, abdominal abscess	CT	5.5, D
	M/24^a^	Colon	Syn	16	7	1	1	1	Generalized peritonitis syndrome	CT	6.3, D
	F/53	Ovary	Syn	39	4	9	1	1	Generalized peritonitis syndrome	reoperation	31.8, D
Septicemia (*n* = 4, 3.8 %)	F/41	Stomach	Met	18	0	5	0	2	*Candida parapsilosis*	CT	14.1, S
	F/40^b^	Stomach	Syn	28	5	9	3	2	*Staphylococcus aureus* and *Candida tropicalis* septicemia	CT	5.5, D
	M/24^b^	Colon	Syn	16	7	1	1	1	Gram-negative bacilli	CT	6.3, D
	M/64	Pseudomyxoma peritonei	Syn	33	5	10	1	2	*Enterococcus faecium* infection	CT	7.0, S
Diarrhea (*n* = 2, 1.9 %)	F/62	Colon	Met	25	6	7	2	3	Over 8 stools per day	CT	6.0, D
	F/64	Colon	Met	24	3	6	2	3	Over 8 stools per day	CT	11.1, D
Acute myocardium infarction (*n* = 1, 0.9 %)	F/73	Colon	Syn	26	6	9	2	1	Grades 5	CT	25.9, D

Common Terminology Criteria for Adverse Events version 4.0

^a^Two patients died of SAE

^b^Different SAEs developed in the same patient

^c^All patients recovered after intervention

*M* male, *F* female, *Syn* synchronous, *Met* metachronous, *CIO* complete intestinal obstruction, *IIO* incomplete intestinal obstruction, *AI* adynamic ileus, *CT* conservative treatment, *D* death, *S* survival

Two patients developed severe diarrhea (grade 3) on postoperative days 6 and 8, respectively, received antidiarrheal therapy, restoration of intestinal flora and electrolytes supplementation therapy, and recovered after 15 and 20 days, respectively. Four patients developed septicemia, two of whom were secondary to above anastomosis leakage, and the other two patients were infected with *Candida parapsilosis*, *Enterococcus faecium* on postoperative days 10 and 9, respectively, received intensified antiseptic treatments, and these two patients completely recovered in about 8 days. The last SAE case developed acute myocardium infraction on postoperative day 2 and the patient died.

## Discussion

CRS + HIPEC as a comprehensive treatment strategy makes the best of surgical resection, locoregional chemotherapy, hyperthermal therapy, and large volume abdominal perfusion washing by CRS to remove the peritoneal and abdominopelvic visible tumor, and the synergistic effects of HIPEC to eradicate residual tumor nodules, micrometastases, and free cancer cells. So far, it is the most effective strategy to treat PC [22]. We have launched experimental [23] and clinical [7, 16] studies to prove the safety and effectiveness of CRS + HIPEC for PC. The Netherlands Cancer Institute has proved in colorectal PC patients the 70 % gain in overall survival (22.4 months in the CRS + HIPEC group *vs. *12.6 months with standard palliative therapy) and recommends that CRS + HIPEC be the standard treatment model for CRC PC patients [22]. Spiliotis has conducted a phase III prospective randomized study in recurrent epithelial ovarian cancer and demonstrated that CRS + HIPEC could prolong OS nearly twice as much as single CRS (26.7 *vs. *13.4 months) [24].

The peritoneal-plasma barrier limits the absorption of large molecule-weight drugs by the peritoneum, ensuring high concentration of drugs in the abdominal perfusion solution. As a result, HIPEC increases the direct cytotoxic effects of chemotherapeutic drugs on peritoneal surface tumors and reduces the systemic side effects. The mechanism of lobaplatin is similar to that of other platinum drugs. Specifically, lobaplatin induces the formation of inter-strand Pt-GG and Pt-AG crosslinks, hindering DNA replication and transcription ultimately inhibiting gene expression in tumor cells [25]. Lobaplatin has shown encouraging broad-spectrum anticancer activity against OC, CRC, GC, lung cancer, breast cancer, and nasopharynx cancer [13–15, 26, 27]. Docetaxel is a semisynthetic complex of the taxane class anti-tumor drugs. It binds to free tubulin, accelerates the assembly of tubulin into stable microtubules, and suppresses microtubule depolymerisation, blocks cell cycle in G-M phase, and inhibits cell proliferation. Docetaxel is also a broad-spectrum cytotoxic drug with potent effects on several malignant solid tumors [28, 29]. A phase I clinical trial demonstrated that lobaplatin combined with docetaxel to treat human solid tumors could exhibit short-term efficacy with a low incidence of SAE [13].

To achieve maximal CRS including organs and peritoneal areas resection, the field of operation should be wide, increasing the risks for major complications. A single-center large sample-size study on 243 PC patients revealed a mortality rate 3 %, SAE rate 43 %, and the SAEs mostly being intra-abdominal collection (40 %), infection (38 %), pleural effusion (37 %), pneumothorax (15.6 %), fistula (11.5 %), ileus (9.5 %), pneumonia (7.4 %), bleeding (5.8 %), perforated viscus (5.8 %), pancreas leak (4.5 %), cardiac arrhythmias (4.1 %), and pulmonary embolism (2.9 %) [30]. In this study, 16 (15.2 %) SAEs occurred in 14 patients, and two of them died during perioperative period, mortality and morbidity rates being 1.9 and 15.2 %, respectively. Compared with literature reports, the SAE rate is relatively lower in our study, possibly due to the following: (1) a designated team performing CRS + HIPEC procedures; (2) the use of broad-spectrum and strong antineoplastic HIPEC drugs (lobaplatin 50 mg/m^2^ + docetaxel 60 mg/m^2^ for 60 min); (3) tension-reduced suture in closing abdominal incision and abdominal wound caring every day to ensure proper wound healing; (4) transfusion of blood, fresh-frozen plasma, cryoprecipitation, and albumin to promptly restore hemostasis; (5) use effective broad-spectrum antibiotics to cover gram-positive, gram-negative, anaerobic, and aerobic bacteria; (6) postoperative ICU monitoring; and (7) good nutritional support and quick restoration of electrolytes balance.

Detailed analysis of the 16 SAEs reveals the following features: (1) more advanced clinical stages, with median PCI 26 (range, 15–39); (2) more complex and difficult operation procedures, with median organ resections, areas of peritonectomy, CC, and operation time being 4 (range, 0–7), 8 (range, 1–10), 2 (range, 1–3), and 660 min (range, 250–760 min), respectively; (3) significantly greater negative impact of SAEs on OS, with median OS of only 12.2 (95 % CI, 9.5–15.0) months for SAEs vs. 31.2 (95 % CI, 20.5–41.9) months for non-SAEs. Therefore, more attention should be paid to selecting LPCI patients for CRS + HIPEC treatment, and intensified perioperative care to minimize the risk of SAEs.

In addition to routine hematological examination, the detection of serum TM was necessary. In this study, 82 patients had elevated serum CEA, CA125, or CA199 levels before operation, and 71 cases (86.6 %) had postoperative TM levels reduced or returned to normal. As serum CEA, CA125, and CA199 levels could reflect tumor invasiveness [31], peritoneal free tumor cells in the ascites [32], and proliferative activity of tumor cells in ascites or primary tumor [33], respectively, the reduction of these TM could provide direct evidence that CRS + HIPEC is effective to control PC.

The median OS for the 100 patients was 24.2 (95 % CI, 15.0–33.4) months, with the 1-, 3-, and 5-year survival rates being 77.5, 32.5, and 19.8 %, respectively. From this study, several lines of evidence could be obtained to support the comprehensive strategy for PC. First, the median disease specific OS could reach 31.2 and 27.5 months for patients with non-neoadjuvant chemotherapy and synchronous PC, but 20.7 and 13.2 months for those with neoadjuvant chemotherapy and metachronous PC. Although such differences did not reach statistical significance, possibly due to follow-up time and sample size, the data do suggest that non-neoadjuvant chemotherapy and synchronous PC could obtain relatively better survival from the therapy. Second, in terms of origin of PC, the PMP and OC PC achieved better OS than the others. In our study, the median OS of OC PC, GC PC, and CRC PC was 34.6, 15.7, and 14.9 months, respectively, similar to the literature reports of 26.7, 12.0, and 13.7 months [7, 16, 24], respectively, suggesting that CRS + HIPEC with lobaplatin and docetaxel to treat PC can achieve acceptable clinical outcome. Third, in terms of PCI, 56 % of the total patients in this study had HPCI, and the median OS was 16.3 (95 % CI, 8.6–24.0) months. The results suggest that HPCI patients could still benefit from CRS + HIPEC rather than the traditional treatment with a poor median survival of less than 6 months. However, it was significantly shorter than the median OS of 46.1 (95 % CI, 10.7–81.5) months for LPCI. Fourth, in terms of CC score, 63 % of PC patients with CC0-1 CRS achieved a median OS of 42.9 (95 % CI, 28.3–57.5) months, much longer than the median OS of 13.6 (95 % CI, 10.6–15.6 months) for CC2-3 patients. Therefore, every effort should be made to maximally reduce the tumor burden. Fifth, adjuvant chemotherapy also plays a key role in the comprehensive therapeutic strategy. Appling IP + SC procedures obtains double effects on PC nodules from a bidirectional approach, which have been proved to improve survival [17, 20]. And 51 % of PC patients with postoperative chemotherapy ≥6 cycles reached median OS of 31.9 (95 % CI, 24.1–39.7) months, much longer than the 14.1 (95 % CI, 9.6–18.6) months for those with chemotherapy <6 cycles. These results again support the notion that LPCI, CC0-1, postoperative chemotherapy ≥6 cycles are independent factors for OS benefit [2, 4, 7, 8, 16, 24].

To our knowledge, there have been three major publications on the combination of lobaplatin and docetaxel in clinical setting [13, 14, 34]. One was the observational study on patients with plural effusion or ascites [34]. The other two was phase I/II clinical trials on the safety and efficacy of the two agents on a variety of solid tumors. A phase I clinical trial demonstrated that lobaplatin (35 mg/m^2^) combined with docetaxel (60 mg/m^2^) to treat human solid tumors (non-small cell lung cancer 11, small cell lung cancer 2, breast cancer 2, gastric cancer 1, endometrial carcinoma 1) could exhibit short-term efficacy (the response rate 7.1 %, the disease control rate 78.6 %) and a low incidence of SAE, such as leukopenia 30 % (grade 3), neutropenia 50 % (grades 3–4) without non-hematological toxicities, with median chemotherapy cycles of four (range, 1–6 cycles) [13]. This phase I study established that the maximal tolerated dose for this chemotherapy regimen is lobaplatin 35 mg/m^2^ combined with docetaxel 60 mg/m^2^, and this regimen demonstrated broad-spectrum anti-tumor properties against lung cancer, breast cancer, endometrial cancer, and gastric cancer. A phase II study evaluated the clinical efficacy and safety of lobaplatin (30 mg/m^2^) combined with docetaxel (75 mg/m^2^) on 39 patients with recurrent or metastatic nasopharygenal carcinoma [14]. This study demonstrated 61.5 % overall response rate, 84.6 % disease control rate, and the median time of progression 10 (95 % CI, 7.3–12.8) months. The most common grade 3/4 toxicities included leucopaenia and neutropenia 17.9 %, anemia 5.1 %, and increased aminotransferase level 2.6 %. This study again demonstrated that this lobaplatin-docetaxel combination is effective against squamous cell carcinoma. A clinical observational study demonstrated that lobaplatin (20–30 mg/m^2^) used in intrapleural or intraperitoneal infusion is a safe and effective treatment for patients with malignant pleural effusion or ascites from colorectal cancer or uterine cancer [34]. Compared with these studies, our study is the first to demonstrate that the lobaplatin-docetaxle regimen used in HIPEC is safe and effective against peritoneal carcinomatosis from colorectal cancer, gastric cancer, ovarian cancer, primary peritoneal carcinoma, and pseudomyxoma peritonei. Our dose (50 mg/m^2^ of lobaplatin and 60 mg/m^2^ of docetaxel) used is comparable to those reported above. And the adverse events were also comparable to the reported results.

## Conclusions

CRS + HIPEC with lobaplatin and docetaxel to treat PC is a feasible procedure with acceptable safety and can prolong the OS of patients with PC from gastrointestinal and gynecological malignancies.

## Abbreviations

CA125: Cancer antigen 125
CA199: Cancer antigen 199
CC: Completeness of cytoreduction
CEA: Carcinoembryonic antigen
CRC: Colorectal cancer
CRS: Cytoreductive surgery
GC: Gastric cancer
HIPEC: Hyperthermic intraperitoneal chemotherapy
HPCI: High PCI
ICU: Intensive care unit
IP: Intraperitoneal chemotherapy
LPCI: Low PCI
MPM: Malignant peritoneal malignancies
OC: Ovarian cancer
OS: Overall survival
PC: Peritoneal carcinomatosis
PCI: Peritoneal cancer index
PMP: Pseudomyxoma peritonei
PPC: Primary peritoneum carcinomatosis
SAE: Serious adverse events
SC: Systemic chemotherapy
TM: Tumor markers
